# Genotype × Environment Interaction and Stability Analysis of Commercial Hybrid Grain Corn Genotypes in Different Environments

**DOI:** 10.3390/life12111773

**Published:** 2022-11-03

**Authors:** Azmi Adham, Mohamad Bahagia Ab Ghaffar, Asmuni Mohd Ikmal, Noraziyah Abd Aziz Shamsudin

**Affiliations:** 1 Industrial Crop Research Centre, Malaysia Agricultural Research and Development Institute (MARDI), Kepala Batas 13200, Pulau Pinang, Malaysia; 2 Department of Biological Sciences and Biotechnology, Faculty of Science and Technology, Universiti Kebangsaan Malaysia, Bangi 43600, Selangor, Malaysia

**Keywords:** grain corn, G × E interaction, GGE analysis, stability analysis

## Abstract

The introduction of superior grain corn genotypes with high and stable yield (YLD) in most environments is important to increase local production and reduce dependency on imported grain corn. In this study, days to tasseling (DT), plant height, and YLD of 11 grain corn genotypes were observed in 10 environments to evaluate the effects of genotype (G), environment (E), and genotype by environment interactions (GEI) using GGE analysis and the stability of genotypes using stability parameters. In each location, grain corn genotypes were arranged in three replications using a randomized complete block design. An analysis of variance showed that all three traits were highly significant toward G and E factors, whereas GEI showed that only DT and YLD were highly significant. Genotype V14 produced the highest YLD of 10,354 kg/ha, followed by V4 (10,114 kg/ha) and V2 (9797.74 kg/ha). These three genotypes also dominated in seven out of 10 tested environments. With regard to stability ranking, genotype V4 was the most stable genotype, with a big gap difference between the second (V14) and third places (V2). Therefore, V14, V4, and V2 were the most promising genotypes because of their great YLD performance and most stable across tested environments, which can be recommended to farmers for high-scale planting.

## 1. Introduction

Grain corn (*Zea mays*) is a primary ingredient in broiler feed in Malaysia, which is used as a protein source in their diet. However, the sources of grain corn are heavily dependent on imported stock because of abandoned grain corn production [1]. According to Nor et al. [2], Malaysia imported four million tons of grain corn in 2018, indicating an increase of 42% compared to 2010. However, no domestic grain corn production was recorded in Malaysia. Furthermore, the currency speculation crisis that affected the Malaysian ringgit resulted in higher expenses with regard to imported grain corn [3]. This situation is a big risk to Malaysian food security. Hence, it will affect the cost of poultry feed, primarily in poultry production, by up to 70% of the total cost [4]. In addition, the COVID-19 pandemic and war between Ukraine and Russia have affected the world food security. Based on the United States Department of Agriculture (USDA) and the Organization for Economic Co-operation and Development (OECD), the pandemic and war has threatened the agricultural sector and created uncertainties in the worldwide stock of corn and vegetable oils, which have increased by nearly 140%. Therefore, the government of Malaysia implemented the “Grain Corn Development Master Plan” in 2018 (which will last until 2032) to promote domestic production and domestic uses up to 30% [2].

Encouraging farmers to plant grain corn is the key factor supporting the government plan. However, an environmental evaluation must be conducted to produce adaptable and high yield (YLD) genotypes because the genotype by environment interaction (GEI) can influence YLD production and other performance variation [5]. Therefore, the selection of site-specific genotypes through multilocation trials (MLT) is necessary to evaluate the stability and YLD performance of grain corn genotypes. The MLT may provide superior genotypes and guidance for breeders in selecting superior genotypes based on YLD performance and stability analysis of genotypes across environments [6]. Several studies have reported that the MLT toward the GEI has discovered five highly potential genotypes in Malaysia and four stable genotypes in Ethiopia, which can be used for further trials to release new varieties through breeding programs [5,7]. Setyawan et al. [8] identified four superior grain corn genotypes being better than or equal to their commercial grain corn variety, BISI 18. These genotypes can be released as the new national superior varieties that can be planted in the maize production center. In addition, some studies in Malaysia and North Western Ethiopia have evaluated established genotypes through MLT to identify suitable environments to create maximum production of grain corn [9,10].

The MLT plays an important role in improving the crop, as it can produce reliable results by evaluating the genotypes in certain periods and in different environments [11]. Furthermore, it can allocate specific and discriminating environments by differentiating the genotype performance within minimal replications [12]. The GEI is considered as the main factor in trials, as it may influence the genetic progress by reducing the interaction between phenotypic and genotypic factors [11]. Consequently, the superior genotypes will have stable performances across environments by minimizing the GEI effect [7]. Thus, this study aimed to select the most promising and stable grain corn genotypes for Malaysia by evaluating 11 tested genotypes in 10 different environments through stability and GEI analyses. The results of this study will be an advantage to farmers and plant breeders, as the information will be useful in selecting a compatible variety within an environment and in preselecting an experimental grain corn breeding program.

## 2. Materials and Methods

### 2.1. Planting Material

A total of 11 commercial grain corn genotypes from four seed companies were used in this experiment (Table 1). All genotypes were imported except for V8, which is a local genotype. V1 was used as a control, as it recorded the highest YLD and stability in previous studies [10]. Meanwhile, the other 10 genotypes were selected on the basis of the recommendations by seed companies.

### 2.2. Test Environment

The MLT was conducted in six locations in peninsular Malaysia in 2019 and 2020. Genotypes were evaluated for two seasons in four locations but only for one season in MARDI Pontian and PPK Labis because of the attack of wild animals, pests, and diseases that affect the amount and quality of the plant. The factors that differentiate each environment included the type of soil, region, planting period, temperature, relative humidity, and total rainfall. In this study, each combination of location and season of planting was considered as one environment to evaluate the genotypic response per season. The agroclimatic condition for each environment is presented in Table 2. Ten selected locations were analyzed by measuring the temperature, humidity, and amount of rainfall. These environmental parameters were provided by the Malaysian Meteorological Department (MET). Based on the mean temperature, these environments had a normal temperature ranging from 25.4 °C to 28.4 °C. The relative humidity recorded (72.5–84.5%) remained in the average level of Malaysia’s humidity. The lowest amount of rainfall was recorded for E9 (MARDI Serdang), whereas the highest rainfall was recorded for E1 (MARDI Bachok).

### 2.3. Agronomic Practices

The experiment was conducted using a randomized complete block design with three replications. The main factor is the grain corn genotype, whereas the subfactor is the environment. The amount of organic fertilizer applied was 3000 kg/ha, whereas lime was applied on the basis of soil pH. Considering the poor nutrient content of Bris soil, a double amount of fertilizer was applied. Meanwhile, for the E6 environment, which contained peat soil, organic fertilizer was not applied because the soil already had a high amount of organic matter. Each genotype plot consisted of seven rows with a length of 5 m for each row, whereas the planting distance was 20 and 75 cm within and between rows, respectively. Two seeds were sown in each hill, but only one seedling was allowed to grow after a week. In reducing the border effects, data were recorded from five central rows of each plot. Compound fertilizer 15:15:15 NPK at 400 kg/ha and urea at 60 kg/ha were applied to each plot at 10 and 30 days after sowing (DAS). The chemical control for pests and diseases was implemented when a disease or an insect infestation was observed in the plot area. At 110–120 DAS, the corn will be harvested when the average cob moisture content is lower than 30%.

### 2.4. Data Collection and Analysis

Two types of data were collected in this study, those being plant morphology and YLD. The data included days to tasseling (DT), plant height (PH), and yield (YLD). These data were obtained at 50–70 DAS (DT), 90 DAS for PH, and the harvesting stage for YLD. DT was recorded by observing the genotype pollens, whereas PH was recorded on the basis of four plants in each genotype. For YLD data, all cobs in the five central rows at each genotype plot were measured. Data measurement included the clean weight of the total cobs and the grain moisture content (14%). The formula for 1-ha YLD data is shown as follows:

$$YLD\;at\;14\%\;\left(kg/ha\right)=\frac{Total\;cob\;weight\;with\;no\;husk\;\left(kg\right)\times 1\;ha}{YLD\;area\;\left(m²\right)}\;$$

YLD area (sample size) = 2.00 m × 2.25 m (3 rows) = 4.50 m^2^

The mean of all parameters and statistical analyses of G, E, and GEI were conducted using Statistical Analysis System version 9.4. Meanwhile, “Genotype by Environment Analysis with R” was used in stability analysis. Stability parameters used in this study included the coefficient of variation (CV), Shukla’s variance (σ²), and Wricke’s ecovalence (W).

## 3. Results

### 3.1. Combined Analysis of Variance (ANOVA) and Variability Study

The combined ANOVA (Table 3) shows that the effects of genotype (G), environment (E), and GEI were highly significant for DT and YLD. However, for PH, the effect of GEI was not significant. We also found that for YLD, G accounted for 74.4% of the total variation, whereas E and GEI accounted for 12.9% and 12.7%, respectively, of the total variation.

On the contrary, a variability analysis showed that the YLD was slightly high in data dispersion around the mean values, and the average of the tested genotype produced a YLD of approximately 9000 kg/ha across the environments. This statement was supported by the CV of 31.3% and mean of 9192.9 kg/ha. With regard to morphology, DT showed the lowest CV of 9.2%, whereas PH showed a CV of 12.1%. The mean data revealed that DT had a mean of 56.6, and PH had a mean of 220.0. Most of the genotypes flowered at 56 DAS with an average height of approximately 218 cm.

Meanwhile, the highest heritability value was recorded for PH at 87.7%, followed by YLD at 75.4% and DT at 52%. Based on these data, all of the traits were found to be more influenced by genetic factors than environmental ones.

### 3.2. Mean Value Comparison of Tested Traits across Environments

The comparison of mean values of DT, PH, and YLD for each genotype across environments is shown in Table 4. For PH, V2, V6, and V1 were the top three genotypes with values of 241.5, 234.9, and 232.0 cm, respectively. In addition, V2 has surpassed the control genotype, V1, by 9.5 cm. V2 remained in the top three for every tested trait and surpassed the control genotype. V4 recorded the shortest DT, followed by V2 and V14. The longest DT was recorded for V3 and V6. In addition, V14 recorded the highest YLD, which was significantly different from the control genotype, V1, followed by V4 with the second highest YLD across the 10 environments. The lowest YLD was recorded for V8.

### 3.3. GGE Biplot

#### 3.3.1. Which-Won-Where/What Biplot

**Figure 1:**
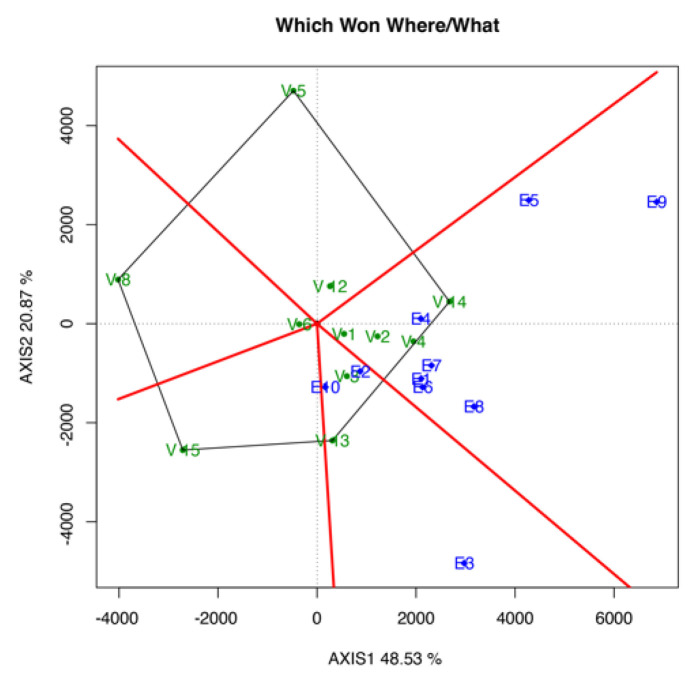
Which-won-where/what biplot for yield indicates the best environment for the tested genotypes.

Figure 1 shows the which-won-where/what polygon pattern, indicating the winning genotypes in each environment. V14 showed the best performance in E4, E1, E6, E7, E8, E5, and E9. Meanwhile, V13 showed the best performance in E2, E10, and E3. Furthermore, V15, V8, and V5 showed a poor performance in all of the tested environments (Figure 1).

#### 3.3.2. Mean vs. Stability Biplot

**Figure 2:**
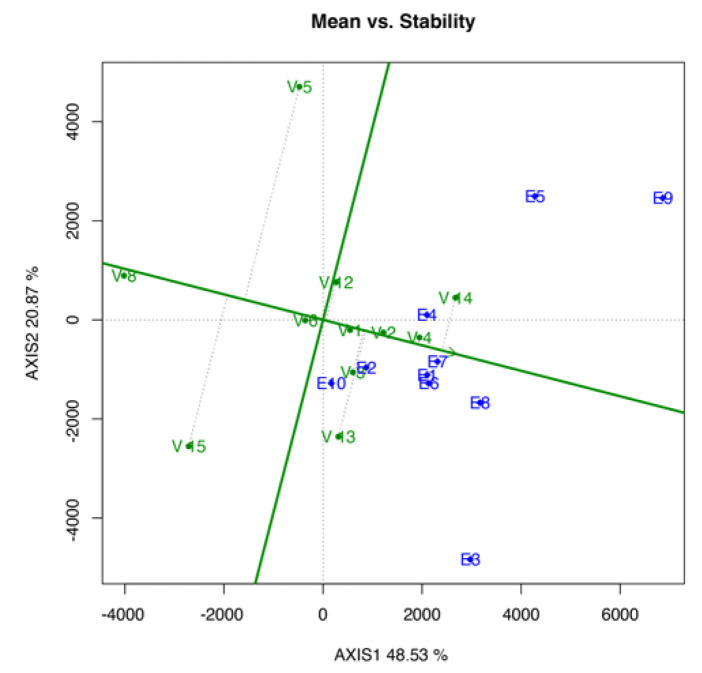
Mean vs. stability biplot analysis, indicating the ranking of the tested genotype based on yield mean and stability.

The mean vs. stability biplot shown in Figure 2 aimed to rank the tested genotypes based on the YLD mean performance and stability. As shown in Figure 2, V1 ranked first as the most stable genotype, followed by V6, V2, V4, V8, V3, V12, V14, V13, V15, and V5. On the contrary, V14, V4, V2, V3, V13, V1, and V12 had higher YLD mean than the overall mean performance, and V6, V5, V15, and V8 had lower YLD mean than the overall mean performance.

#### 3.3.3. Ranking Genotype Biplot

**Figure 3:**
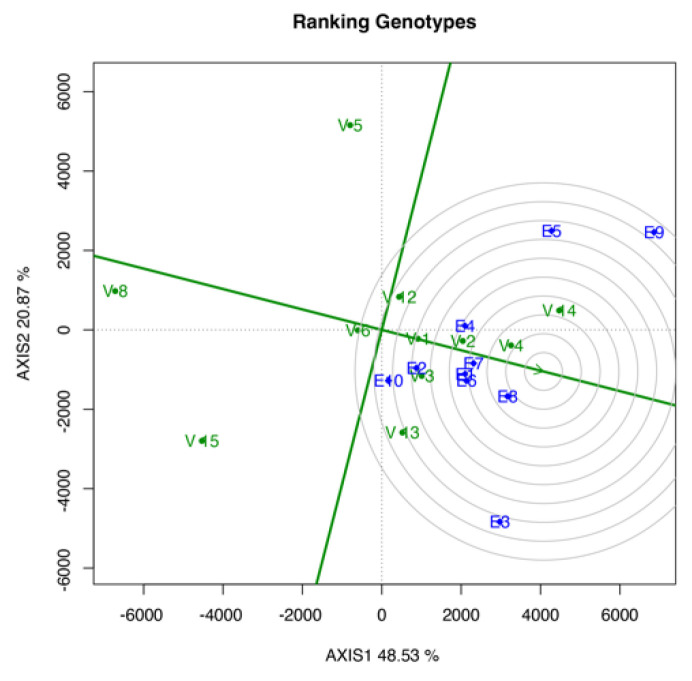
Ranking genotype biplot shows the best genotype performance in yield mean across environments.

Figure 3 shows the genotype ranking based on the YLD performance in the tested environment. V4 ranked first because it was the closest to the innermost circle, followed by V2 and V14. Meanwhile, V8, V5, and V15 ranked in the bottom three, as these genotypes were the furthest from the innermost circle. As shown in Figure 3, the genotype ranking from top to bottom was as follows: V4, V14, V2, V3, V1, V13, V12, V6, V5, V15, and V8.

#### 3.3.4. Discriminativeness vs. Representativeness Biplot

**Figure 4:**
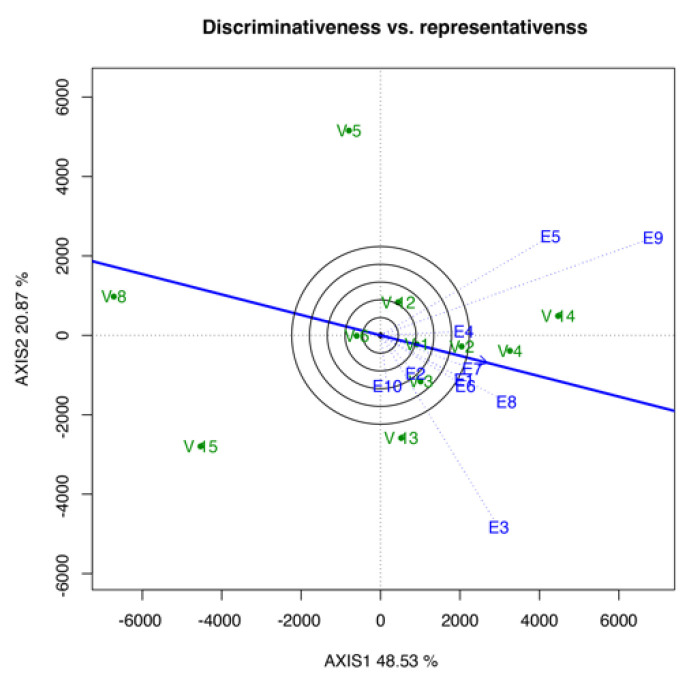
Discriminativeness vs. representativeness of tested environments toward yield mean.

Discriminativeness versus the representativeness of the environment is shown in Figure 4. This biplot was used to determine the discriminative ability of the environment based on the length of the environmental vector. Therefore, among the 10 studied environments, E9 exhibited the most discriminating environment, followed by E3, E5, and E8, whereas E2 was the least discriminating. As shown in Figure 4, environment E7 was the most representative environment, followed by E8, E1, and E6.

#### 3.3.5. Ranking Environment Biplot

**Figure 5:**
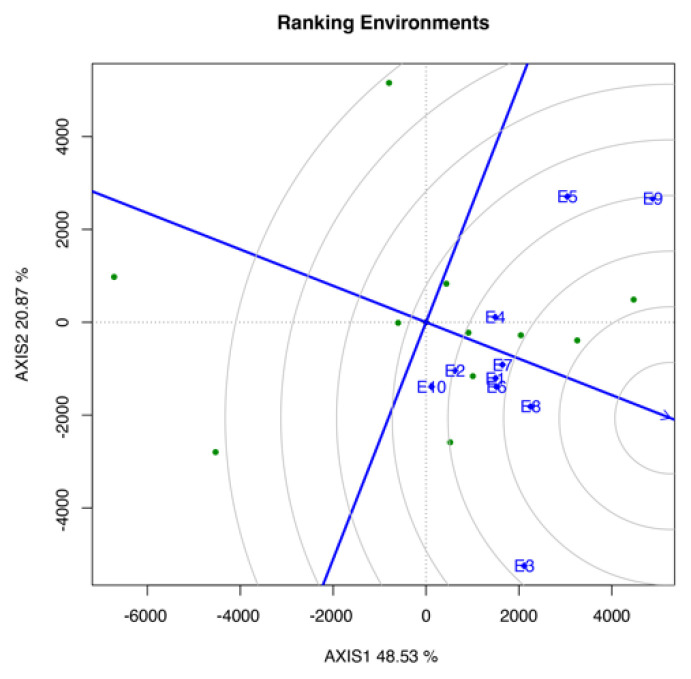
Environment ranking was determined by comparing the “ideal environment” with yield mean.

The ideal environment was located at the center of the concentric circles, which served as the most discriminating representative of the target environment (Figure 5). Thus, environment E8 was the closest environment to the ideal environment. As shown in Figure 5 the ranking from top to bottom was as follows: E8, E6, E7, E1, E4, E3, E2, E9, E10, and E5.

#### 3.3.6. Stability Statistical Parameters

Table 5 shows the stability statistical parameters among the tested genotype and environments. These parameters were based on the smallest values to determine the best stability among genotypes. Based on Francis cumulative values, V1 had the smallest value, followed by V4 and V13, accounting for 16.7, 18.0, and 19.3 CV respectively. Meanwhile, based on Shukla stability variance (σ^2^) and Wricke’s ecovalance (W), V12 had the lowest values, followed by V6 and V4, whereas V15 had the highest values. Therefore, V4 showed the best stability among the genotypes, as it was in the top three of these stability parameters.

## 4. Discussion

In the present study, 11 hybrid grain corn genotypes were evaluated. In the total variation partitioning, the higher percentage of variation for GEI compared with G indicates the large difference in the performance of genotypes across the tested environments. According to Yan and Kang [13], when a significant amount of variation was detected for GEI, the existence of different mega-environments was inferred, where different genotypes would have the best YLD in each of the mega-environments.

ANOVA (Table 3) revealed the significant effect of GEI on DT and YLD, which indicated the need for further evaluation. Meanwhile, for PH, as the effect of GEI was not significant, mean comparison analysis was sufficient to detect the differences among genotypes across environments. For DT and YLD, GGE biplot analysis was performed to obtain more information for the selection of superior genotypes [14]. However, considering that the YLD was the main focus of this study, only YLD traits were used in these analyses.

A heritability analysis aimed to investigate the relationship between phenotypic and genotypic values toward environmental factors. In addition, it was also used to improvise the genotypic selection based on the phenotypic performance [15]. In this study, the genotypic selection was based on the phenotypic performance that has a high heritability value, which was influenced by the additive gene action and could improvise the genotypic selection [16]. The high heritability values for all tested traits indicated that such traits were affected greatly by environmental factors; thus, the genotypic selection based on the phenotypic performance became reliable and effective [17]. In addition, according to Syafii et al. [18], the quantitative traits that contained high heritability values can be used as a selection parameter for producing a new genotype. Furthermore, Amzeri and Badami [19] stated that high heritability values can be directed to assemble pure line and hybrid varieties. On the contrary, the ANOVA results showed a high significance of DT and YLD toward GEI, which provided positive support through genotypic selection. This finding may lead to the identification of “ideal” genotypes that contain the highest mean performance and heritability values among tested genotypes [20]. In addition, it revealed the ideal environment that provided the most discriminative and representative abilities [21]. Therefore, analyses of GEI and GGE biplots could make a specific and accurate selection, evaluation, and identification of ideal genotypes for multienvironments [22]. Moreover, GEI analysis determined the ideal environment that effectively identifies the superior genotype [23].

The which-won-where biplot pattern is determined by relative genotypic stabilities and the average performance of genotypes in tested environments. According to Yan and Rajcan [24], the pattern in the biplot can also explain the presence or absence of crossover in GEI. The existence of a mega-environment can also be inferred on the basis of the biplot pattern. In the present study, a crossover type of GEI was detected, as different genotypes won or performed best in different environments. Given the presence of crossover, more than one mega-environment was detected.

All of the tested environments recorded values of temperature and relative humidity that were within the normal range. However, a large difference was found in the amount of total rainfall, particularly between E1 and E9, with a difference of 989 mm. According to Kang and Gorman [25], the interaction between genotypes and environments will lose 1.4% sum of squares if there was rainfall during the growing season and lose 1.1% sum of squares of heterogeneity if it was a preseason rainfall. Based on the environment ranking biplot (Figure 5), E8, E6, and E7 were the top three, with total rainfall of 375.9, 384.3, and 113.4 mm, respectively. Meanwhile, E1, which recorded the highest amount of total rainfall, only ranked fourth. Therefore, the high total rainfall may affect the flowering process, thereby causing a low YLD production.

In addition, these tested environments contain peat, Bris, and mineral types of soil. These different types of soil may become the subfactor to evaluate the environmental performance, as each type of soil contains a different character. As for Bris soil, its sandy texture and low soil fertility affected fertilizer application, which may be lost from the soil system and cause pollution and negative effects on the surrounding water environment [26]. Thus, E1 and E2 received two times the amount of organic fertilizers. For peat soil, it was formed from the decays of accumulated organic matter, which then produced low pH, different particle-size distribution from inorganic soils, and high organic and water content, making it a good physical medium for annual crops [27]. On the contrary, mineral soil has a fine texture that can retain sufficient moisture and excellent drainage, which is good for root penetration if no stones or laterite is found in its profile, but its lack of phosphorus leads to the need for the regular application of fertilizer [28]. Given these soil characteristics, the type of soil may become the subfactor affecting the YLD performance of the tested genotypes.

In selecting the ideal test environment, it has to be discriminative and representative of the genotypes and mega-environment [23]. If an environment has a high capacity for genotypic discrimination and representativeness, then it is an ideal environment. Based on the discriminativeness and representativeness biplot (Figure 4), most of the environments cannot hold the top position in both analyses, such as E9. This environment was the most discriminating but not the most representative. Similar to E7, it was the most representative environment but not the most discriminative. Meanwhile, only E8, which holds the top four positions in both analyses, makes the nearest toward the “ideal” environment to evaluate an adapted genotype [29]. This result was supported by the environment ranking analysis (Figure 5), which stated that E8, E6, and E7 were the best three environments for the tested genotype based on the YLD mean performance. This analysis also showed that E8 was the best environment, providing suitable temperature, humidity, and total rainfall, which causes the genotypes to perform better than in other environments.

The ranking in the mean *vs*. stability biplot (Figure 2) can be measured by referring to the green line with a single arrowhead, which serves as the average environment coordinate (AEC) abscissa. The genotype closest to the green line will be established as the most stable genotype, whereas those further away will become more variable and less stable across the environment [30]. The highest and lowest mean performance of genotypes is defined by referring to the vertical lines passing the origin perpendicular to the AEC abscissa. In ranking the genotypes using the dry YLD mean, the arrowhead pointing toward the right is referred. Based on the results, the control genotype (V1) was the most stable genotype among the other genotypes. However, only a slight gap difference was observed among the top four positions. V6, V2, and V4 were the top four genotypes. Meanwhile, genotypes V13, V15, and V5 were not selected because of their gap from the green line. In the overall mean performance, V1 ranked sixth, whereas V14 and V4 had the highest mean YLD. The ranking genotype biplot was based on the distance between the genotypes and the ideal genotype that is located at the center or the innermost circle. An ideal genotype should have the highest mean performance and great stability [30]. Evidently, V4 was the closest genotype to the ideal genotype, which was found at the third circle layer, followed by V14 and V2 at the fourth and fifth circle layer, respectively. Meanwhile, the control genotype, V1, was found at the seventh layer of the circle from the innermost circle, placing it in fifth place in the overall genotype ranking.

According to Shukla [31] and Wricke [32], the most stable genotypes are those with the lowest stability variance. Meanwhile, as stated by Francis and Kannenberg [33], genotypes that exhibit low CV and high YLD are considered stable. Therefore, the selection of superior genotypes must be based on the combination of stability and YLD performance. Although V12 has good values of stability parameters, the recorded YLD was lower than that of V4, V14, and V2.

Combining these analyses, V4, V14, and V2 were the best genotypes based on their genotype ranking, stability, and YLD mean performance. Although these genotypes were the best in this study, performing a validation test on farmer’s farm before promoting this genotype for commercial farming is recommended. Thus, the results will provide specific data which may minimize Malaysia’s rate of grain corn imports and indirectly support the government’s plan to implement the Grain Corn Development Master Plan.

## 5. Conclusions

Based on the results and discussion, V4 becomes the most appealing genotype in overall tested traits, which has dominated the top two values in DT, YLD mean, and stability rankings. Moreover, V4 shows great performance in most of the tested environments and becomes the closest genotype to the ideal genotype. Furthermore, V14 and V2, which were included in the top three in the genotype rankings, can be the second option in promoting the genotype. The best environment to conduct domestic grain corn production was in the north of peninsular Malaysia, which was represented by E8, which dominated the environment ranking biplot. E9 was found to be the most suitable environment to conduct research because it had the most discriminating environment. Based on the analysis, these winning genotypes and environments should be recommended to farmers to achieve the objectives of the government programs and indirectly reduce the country’s imported rate of grain corn. Moreover, these winning genotypes and environments should be included for further trials of grain corn in MLTs.

## Figures and Tables

**Table 1:** List of tested genotypes and their information.

Variety	Seed Producer	Country of Origin	Code
P 4546	Pioneer	Thailand	V1
P 3875	Pioneer	Thailand	V2
P 3582	Pioneer	Thailand	V3
P 4554	Pioneer	Thailand	V4
P 3537	Pioneer	Thailand	V5
P 3136	Pioneer	Thailand	V6
GWG 888	Green World Genetics	Malaysia	V8
GT 722	Golconda	Thailand	V12
GT 822	Golconda	Thailand	V13
DK 9979 C	Monsanto	Thailand	V14
DK 9950 C	Monsanto	Thailand	V15

**Table 2:** Agroclimatic conditions of the selected environments.

Env	Location	Coordinate	Region	Soil Type	Planting Period	Mnt (°C)	Mxt (°C)	Met (°C)	RH (%)	TR (mm)
E1	MARDI Bachok	5.97838, 102.42752	East	Bris	Aug–Dec 2019	25.9	27.9	27.0	82.2	1078.8
E2	MARDI Bachok	5.97838, 102.42752	East	Bris	Jul–Nov 2020	26.0	28.1	27.1	83.4	715.0
E3	MARDI Kluang	1.942824, 103.356674	South	Mineral	Aug 2019–Jan 2020	25.4	27.5	26.6	81.2	280.1
E4	MARDI Kluang	1.942824, 103.356674	South	Mineral	Feb–Jun 2020	26.2	28.2	27.4	83.5	734.4
E5	PPK Labis	2.3743913, 103.0151	South	Mineral	Aug 2019–Jan 2020	24.2	26.3	25.4	76.5	607.6
E6	MARDI Pontian	1.50673592857, 103.446522947	South	Peat	Feb–Jun 2020	25.9	27.9	27.0	79.5	384.3
E7	MARDI Seberang Perai	5.5399872, 100.4700515	North	Mineral	Dec 2019–May 2020	27.7	29.0	28.4	72.5	113.4
E8	MARDI Seberang Perai	5.5399872, 100.4700515	North	Mineral	Aug–Dec 2020	26.0	27.6	26.8	84.5	375.9
E9	MARDI Serdang	2.926361, 101.696445	West	Mineral	Jan–May 2020	27.5	28.6	28.0	75.6	89.8
E10	MARDI Serdang	2.926361, 101.696445	West	Mineral	Jul–Nov 2020	26.2	28.2	27.3	80.3	391.4

Mnt, minimum temperature; Mxt, maximum temperature; Met, mean temperature; RH, relative humidity; TR, total rainfall.

**Table 3:** Analysis of variance (mean square values), mean, CV, and heritability for days to tasseling (DT), plant height (PH), and yield (YLD) across 10 environments.

Sources	DF	DT			PH			YLD		
		SS	MS	%SS	SS	MS	%SS	SS	MS	%SS
Env (E)	9	6770.1	752.2 **	86.3	53337.0	5926.3 **	34.5	1381818260	153535362 **	74.4
Rep (Env)	20	347.6	17.4 **		19478.9	973.9 **		239091700	11954585 **	
Gen (G)	10	147.9	14.8 **	1.9	66337.2	6633.7 **	42.9	235618383	235618383 **	12.9
G × E	90	924.5	10.3 **	11.8	35067.1	389.6 ns	22.7	404422753	4493586 **	12.7
Error	199	691.7	3.5		59116.6	297.1		459032793	2306697	
Mean ± S.E.			56.6 ± 0.3			220.0 ± 1.5			9192.9 ± 158.6	
CV (%)			9.2			12.1			31.3	
Heritability (%)			52.0			87.7			75.4	

ns, not significant; ** significant at 0.01 probability level.

**Table 4:** Mean value comparison of tested traits for 11 genotypes across environments.

Genotype	DT (days)	PH (cm)	YLD (kg/ha)
V1	57.0 ^abc^	232.0 ^bc^	9451.8 ^bc^
V2	55.7 ^d^	241.5 ^a^	9797.7 ^abc^
V3	57.5 ^a^	227.2 ^bc^	9648.7 ^abc^
V4	55.6 ^d^	223.9 ^cd^	10,114.0 ^ab^
V5	56.5 ^bcd^	228.0 ^bc^	8455.2 ^d^
V6	57.5 ^ab^	234.9 ^ab^	8998.0 ^cd^
V8	56.3 ^cd^	197.5 ^e^	7214.7 ^e^
V12	57.4 ^ab^	201.4 ^e^	9255.3 ^bcd^
V13	56.4 ^bcd^	201.0 ^e^	9353.7 ^bc^
V14	55.8 ^d^	217.7 ^d^	10,354.0 ^a^
V15	56.4 ^bcd^	215.2 ^d^	8471.5 ^d^

Mean values with different letters are significantly different (Duncan’s Multiple Range Test).

**Table 5:** Stability statistical parameters.

Gen	Mean	Francis (CV)	GR	Shukla (σ²)	GR	Wricke’s Ecovalence (W)	GR
V1	9451.8	16.7	1	1069303	4	9102252	4
V2	9797.7	24.3	5	1133867	5	9577677	5
V3	9648.7	32.5	9	1469220	8	12047096	8
V4	10,114.2	18.0	2	680575.8	3	6239804	3
V5	8455.2	31.5	8	2952960	10	22972815	10
V6	9058.6	24.5	7	494521.2	2	4869765	2
V8	7214.7	38.7	10	1413761	6	11638714	6
V12	9255.3	23.0	4	238061	1	2981286	1
V13	9353.7	19.3	3	1645989	9	13348753	9
V14	10,354.0	24.5	6	1446722	7	11881423	7
V15	8471.5	39.3	11	3968710	11	30452429	11

GR, genotype ranking.

## Data Availability

The data presented in this study are available on request from the corresponding author. The data are not publicly available due to restriction of company procedure.
